# Cryptic exon detection and transcriptomic changes revealed in single-nuclei RNA sequencing of *C9ORF72* patients spanning the ALS-FTD spectrum

**DOI:** 10.1007/s00401-023-02599-5

**Published:** 2023-07-19

**Authors:** Lauren M. Gittings, Eric B. Alsop, Jerry Antone, Mo Singer, Timothy G. Whitsett, Rita Sattler, Kendall Van Keuren-Jensen

**Affiliations:** 1 Department of Translational Neuroscience, Barrow Neurological Institute, 350 W Thomas Road, Phoenix, AZ 85013 USA; 2 Neurogenomics Division, Translational Genomics Research Institute, part of City of Hope, Phoenix, AZ USA

**Keywords:** *C9ORF72*, Single nuclei RNA sequencing, TAR DNA-binding protein 43 (TDP-43), Cryptic exon (CE), Amyotrophic lateral sclerosis (ALS), Frontotemporal dementia (FTD)

## Abstract

The *C9ORF72*-linked diseases amyotrophic lateral sclerosis (ALS) and frontotemporal dementia (FTD) are characterized by the nuclear depletion and cytoplasmic accumulation of TAR DNA-binding protein 43 (TDP-43). Recent studies have shown that the loss of TDP-43 function leads to the inclusion of cryptic exons (CE) in several RNA transcript targets of TDP-43. Here, we show for the first time the detection of CEs in a single-nuclei RNA sequencing (snRNA-seq) dataset obtained from frontal and occipital cortices of *C9ORF72* patients that phenotypically span the ALS-FTD disease spectrum. We assessed each cellular cluster for detection of recently described TDP-43-induced CEs. Transcripts containing CEs in the genes *STMN2* and *KALRN* were detected in the frontal cortex of all *C9ORF72* disease groups with the highest frequency in excitatory neurons in the *C9ORF72*-FTD group. Within the excitatory neurons, the cluster with the highest proportion of cells containing a CE had transcriptomic similarities to von Economo neurons, which are known to be vulnerable to TDP-43 pathology and selectively lost in *C9ORF72*-FTD. Differential gene expression and pathway analysis of CE-containing neurons revealed multiple dysregulated metabolic processes. Our findings reveal novel insights into the transcriptomic changes of neurons vulnerable to TDP-43 pathology.

**Supplementary Information:**

The online version contains supplementary material available at 10.1007/s00401-023-02599-5.

## Introduction

Nuclear depletion and cytoplasmic aggregation of the TAR DNA-binding protein 43 (TDP-43) is a pathological hallmark of amyotrophic lateral sclerosis (ALS) and frontotemporal dementia (FTD) patients caused by a hexanucleotide repeat expansion mutation in the *C9ORF72* gene (C9-ALS and C9-FTD) [35, 37, 43]. It is also the primary pathology seen in sporadic ALS, most familial forms of ALS (with the exception of *FUS* and *SOD1* mutations), and 50% of sporadic and familial FTD cases [47, 48], and has also been described in other neurodegenerative diseases, such as Alzheimer’s disease [40, 45]. While in ALS with TDP-43 inclusions (ALS-TDP), TDP-43 pathology is primarily observed within neurons and glia in the spinal cord, motor cortex and brainstem motor nuclei [34], the distribution and morphology of TDP-43 inclusions in FTD with TDP-43 inclusions (FTD-TDP), particularly in *C9ORF72* mutation carriers, is more heterogenous [35, 36, 46]. It is currently unknown if there are cell types within the cortex that are more susceptible to TDP-43 mislocalization.

TDP-43 is a DNA/RNA binding protein that performs a variety of functions associated with RNA metabolism including transcription, splicing, transport, and stabilization [51]. A recently described phenotype governed by the mislocalization of TDP-43 in ALS-TDP and FTD-TDP is a failure of TDP-43 to repress the inclusion of cryptic exons (CEs) in several RNA transcript targets of TDP-43 [4, 23, 24, 29, 33, 39, 50]. The inclusion of CEs within these transcripts can introduce a frameshift or premature stop codons, destabilize the mRNA, resulting in aberrant transcripts being targeted for nonsense-mediated decay (NMD), or code for aberrant peptides that might have deleterious effects if produced [1, 9, 23].

Two well-characterized CEs that occur as a result of the loss-of-function of TDP-43 in ALS-TDP and FTD-TDP are in the genes *STMN2* and *UNC13A* [4, 24, 33, 39, 50]. For *STMN2*, loss-of-function of TDP-43 results in the inclusion of a CE, exon 2a, between exon 1 and exon 2. This introduces a premature stop codon and polyadenylation signal into the *STMN2* mRNA transcript [2, 24, 39, 50]. A similar phenomenon occurs in *UNC13A* transcripts as a CE gets included in *UNC13A* mRNA between exon 20 and exon 21 upon loss-of-function of TDP-43. This cryptic splicing event results in a decrease in *UNC13A* mRNA and protein, likely because the mRNA is degraded by the process of NMD [1, 4, 33]. The CEs in both *STMN2* and *UNC13A* transcripts were detected by bulk RNA sequencing in postmortem tissue from patients with ALS-TDP and FTD-TDP, and their detection is considered a marker for TDP-43 pathology [4, 50]. In addition to *STMN2* and *UNC13A*, several other genes have demonstrated alternative splicing due to a loss-of-function of TDP-43, including several genes involved in synaptic function, such as *KALRN*, *RAPGEF6* and *SYT7* [23, 29, 33, 54, 63].

Despite being able to detect TDP-43-induced CEs in patient post-mortem tissue, thus far it has not been possible to determine which specific cell types these CEs originate from. Understanding the cellular origin of these CEs will provide a better insight into the cell types that are vulnerable to TDP-43 pathology. This is of particular interest in FTD-TDP where the cell types of the frontal cortex that are impacted by TDP-43 pathology are less well-defined than the cells of the spinal cord, motor cortex and brainstem motor nuclei that are known to be susceptible to TDP-43 pathology in ALS-TDP.

In this study, we performed single-nuclei RNA sequencing (snRNA-seq) on the frontal cortex of *C9ORF72* mutation carriers that spanned the ALS-FTD phenotypic spectrum (C9-ALS, C9-ALS/FTD, C9-FTD) to identify individual cells that contained CEs in known splicing targets of TDP-43. CEs in the transcripts of *STMN2* and *KALRN* were detected in multiple neuronal subtypes, as well as non-neuronal cell types, in all *C9ORF72* disease groups in a manner reflective of the expected level of TDP-43 pathological burden in the frontal cortex for each group (C9-FTD > C9-ALS/FTD > C9-ALS). Furthermore, we determined that L5 extra telencephalic neurons were susceptible to TDP-43 pathology by quantifying the number of cells containing CEs. When we analyzed the transcriptome of CE-containing cells, genes in the NMD pathway were dysregulated, a known cellular pathway involved in the clearance of some CE-affected mRNAs. This data provides a novel perspective on the cell type and number of cells with detectable TDP-43-induced CEs in *C9ORF72* patients and provides insight into the transcriptional changes in these cells.

## Methods

### Postmortem tissue samples

Frozen, post-mortem frontal and occipital cortex tissue samples from *C9ORF72* patients and control donors without neurological disorders were obtained from the Target ALS Human Postmortem Tissue Core, the Queen Square Brain Bank for Neurological Disorders and the Banner Sun Health Research Institute Brain and Body Donation Program. Individuals were properly consented to participation at each biorepository. Disease groups were determined based on the pathological diagnosis of disease provided by the brain banks (ALS only, FTD only, ALS-FTD and Control). Demographic information on the tissues used in this study is provided in Supplementary Table 1.

### Isolation and purification of nuclei

Frozen frontal cortex tissue (50 mg) was dounce homogenized in 1 mL of Nuclei Lysis buffer [Nuclei EZ Lysis Buffer (Sigma-Aldrich, St. Louis, MO, USA), 1 × cOmplete Protease Inhibitor Cocktail (Sigma-Aldrich, St. Louis, MO, USA), and RNasin Plus (Promega)] 10–15 times using pestle A “loose” followed by pestle B “tight'' 10–15 times (DWK Life Sciences, Millville, NJ, USA). Homogenate was passed through a 70 µm 1.5 mL mini strainer (PluriSelect, El Cajon, CA, USA) and centrifuged at 500 rcf for 5 min at 4 °C. Nuclei pellet was resuspended in an additional 1 mL of Nuclei Lysis buffer and incubated for 5 min followed by centrifugation at 500 rcf for 5 min at 4 °C. 500 µl of 1 × wash buffer (1 × PBS, 2% BSA, 0.2 U/µl RNasin Plus) is added to the nuclei pellet and incubated for 5 min to allow adequate buffer exchange followed by centrifugation at 500 rcf for 5 min at 4 °C, repeated once more and resuspend in 500 µl of 1 × wash buffer. Resuspended nuclei were incubated in 1–2 drops of NucBlue Live ReadyProbes Reagent (ThermoFisher Scientific) and immediately sorted using the DAPI channel on the Sony SH800S (Sony Biotechnology, San Jose, CA, USA) with a 100 µm chip.

### 10x Genomics snRNA-seq and sequencing

Nuclei were sorted for 15,000 events directly into 10 × 3´ v3 RT Reagent Master Mix and immediately processed with the 10x Genomics Chromium Next GEM Single Cell 3´ v3 kit (10x Genomics, Pleasanton, CA). To minimize batch effects, each 10x chip contained samples from all disease and control groups. Samples were loaded, cDNA amplified, and library constructed following the manufacturer’s protocol. Library quality control (QC) was based on Agilent TapeStation 4200 HS D1000 screentapes (Agilent Technologies, Waldbronn, Germany). Multiplexed library pool was based on Agilent TapeStation 4200 HS D1000 and Kapa Library Quantification Kit for Illumina platforms (Kapa BioSystems, Boston, MA) and sequenced at shallow depths on Illumina’s iSeq 100 v2 flow cell for 28 × 8x91 cycles for estimated reads per cell. After demultiplexing, libraries were rebalanced based on reads per cell. Normalized pool QC was based on Agilent TapeStation 4200 HS D1000 and Kapa Library Quantification Kit for Illumina platforms and high depth sequenced on Illumina’s NovaSeq 6000 S4 v1.5 flow cell for 28 × 8 × 91 cycles.

### SnRNA-Seq quality control filtering and normalization

BCL files from NovaSeq S4 flowcells were processed using Cell Ranger v. 5.0.1 (10x Genomics) using cell ranger mkfastq to make fastq files for each sample [61]. Each sample was then processed with cell ranger count using the human reference database provided by 10x Genomics (gex-GRCh38-2020-A) using the –include-introns option, as recommended for single-nuclei data. All resulting filtered counts matrices (filtered_feature_bc_matrix files) for all samples were loaded into scanpy and combined into a single gene expression matrix using concatenate function [57]. Resulting data object was then filtered to remove any nuclei with > 5% ribosomal genes, > 5% mitochondrial genes, > 0.1% hemoglobin genes and less than 200 total genes. The highly expressed and variable lncRNA MALAT1 was removed from the data along with all ribosomal (RPL & RPS), mitochondrial (MT-) and hemoglobin (HB-) genes. Genes not expressed in at least 3 nuclei were also removed. Doublets were then identified and removed using scrublets [58]. The resulting, quality filtered, data matrix was normalized using the computeSumFactors function from the scran R package [31]. This package performs a scaling normalization of single-cell RNA-seq data by deconvolving size factors from cell clusters. Following size factor normalization, the counts data were logarithmized using the log1p function in scanpy and the resulting log-transformed gene counts data was stored as a data object for downstream analysis.

### Batch correction and cluster generation

To facilitate accurate cluster determination, batch correction was performed using scanorama [19] between samples (batch_key = 'sample') on the top 4000 most highly variable genes, as determined using highly_variable_genes(flavor = 'cell_ranger') with the integrate_scanpy() function. Principal component analysis (PCA) and nearest neighbor calculations were performed using batch-corrected data (n_neighbors = 50, n_pcs = 50, use_rep = "Scanorama"), followed by uniform manifold approximation and projection (UMAP) generation. Graph clustering was performed using the Leiden algorithm in scanpy with a cluster resolution of 0.3. Samples from some subjects were prepared more than once to balance sample loading on the 10x Genomics chips, to provide equal numbers of males, females or diseases and controls with each run. In this case, samples from the same subject were combined by subject ID (Supplementary Table 1, online resource).

### Cluster cell type annotation

Leiden clusters were annotated using established marker genes from cortex samples by analyzing dot plots and UMAPs depicting the expression levels of marker genes found in each Leiden cluster. The following marker gene list was used: *AQP4* and *GFAP* (astrocytes), *EPAS1* and *CLDN5* (endothelial cells), *RBFOX3* (neurons), *SATB2*, *SLC17A7* and *NRGN* (excitatory neurons), *GAD1* and *GAD2* (inhibitory neurons), *ADAM28* and *APBB1IP* (microglia), *RNF220* and *ST18* (oligodendrocytes), *PDGFRA* and *SMOC1* (oligodendrocyte progenitor cells; OPCs). This resulted in one astrocyte cluster, one endothelial cell cluster, eight excitatory neuron clusters, four inhibitory neuron clusters, one microglia cluster, one oligodendrocyte cluster and one OPC cluster. In the frontal cortex, we were able to further define the excitatory and inhibitory neuron clusters using marker genes [3, 21, 32, 56]. The following marker gene list was used: *SNAP25* (neuron marker), *SATB2* (excitatory neurons), *CUX2*, *PCDH8* and *CCDC88C* (L2–L3 intratelencephalic), *RORB*, *TWIST2* and *ALDH1A1* (L3–L5 intratelencephalic type 1), *PKD2L1* and *ABCC12* (L3–L5 intratelencephalic type 2), *HTR2C*, *ADAMTS12* and *NPSR1* (L5-L6 near projecting), *POU3F1*, *ABCB11* and *SLC66A1L* (L5 extratelencephalic), *OPRK1* (L6 intratelencephalic type 1), *SMYD1* and *SNTB1* (L6 intratelencephalic type 2), *SYT6*, *CTGF*, and *GALR1* (L6 corticothalamic / L6B), *GAD1* (inhibitory neurons), *SST*, *HGF* and *LHX6* (somatostatin interneurons), *PVALB* and *CALN1* (parvalbumin interneurons), *VIP* and *NR2F2-AS1* (VIP interneurons), *SV2C* and *LAMP5* (SVC2 LAMP5 interneurons). Differences in cell type abundance between disease groups were calculated using stat_compare_means() in R using the Wilcoxon test method.

### Cryptic exon junction analysis

Junctions were identified as described in Brown et al*.* [4]. Briefly, fastq files were aligned to the human genome (GRCh38) using STAR with ENCODE standard alignment parameters. Resulting BAM files were then analyzed with regtools junctions extract (options: -a 6 -m 30 -M 500000) to generate junction files, which include novel junctions, for all samples [5]. Leafcutter’s leafcutter_cluster_regtools.py (options: -m 10 -p 0.0001) was then used to generate a summary counts matrix of all junctions found in the dataset [28]. For single-nuclei data, each sample was aligned to the human genome using cellranger counts (described above) and each sample’s BAM files were split into multiple cell-type specific BAM files using each cell’s 10x gem barcode and the cluster annotation for that barcode, as determined above. Further, nuclei-level BAMs were generated by parsing each sample’s BAM file and splitting by 10x barcodes to generate one BAM file per barcode. Each per-sample cell-type and nuclei-specific BAM file was then processed independently through regtools (using the above parameters) and all resulting junction files were collapsed using leafcutter (using the above parameters) to generate a summary counts matrix of all junctions found in all individual nuclei. A list of 66 alternatively spliced genes due to TDP-43 mislocalization was recently published [33]. Using this list of 66 genes and the NeuN(+) TDP-43(+) and TDP-43(–) nuclear sequencing data from Lui et al. [30], we identified genomic coordinates and trained our detection pipeline for CEs using the methods outlined above starting with regtools. We selected junctions that were found to have an average of > 10 counts in TDP-43 negative samples, and a fold change > 20 when compared to matched TDP-43 positive samples in the data. We then tested these genomic coordinates in the single nuclei data, and a junction was considered detected in the 10x Genomics data if it was found at an average expression level > 0.2 in *C9ORF72* samples with a fold change > 10 when compared to matched controls. CE junctions were defined by their overlap between those found in our single-nuclei data and those in our re-analysis of the data from Lui et al. [30]. This resulted in two, well-detected CE junctions (*STMN2* chr8:79,611,214–79,616,822 and *KALRN* chr3:124,701,255–124,702,038).

### Cryptic exon differential gene expression and pathway analysis

For identified *STMN2* or *KALRN* CEs, we subset the single-nuclei expression data to only C9-FTD samples with a detected CE, and only to excitatory neuron clusters with a detected CE. From this subset of nuclei we used normalized, log-transformed genes count matrices to perform differential expression within each cluster between cells with and cells without an *STMN2* or *KALRN* CE using MAST with the covariate formula: ~ CE_YN + sex + number_of_genes_in_the_cell [11]. Multiple corrections testing (FDR) using the Hurdle Model was implemented to give the adjusted p-values reported. Differentially expressed genes lists from each cluster were subset to genes with a p-value < 0.05 and abs(log2FC > 0.1) and analyzed using the Gene Ontology Biological Process (GOBP) pathway database in clusterProfiler [60]. The pathways with the lowest q-value were subjected to Gene Set Enrichment Analysis (GSEA), performed using the fgsea package in R using the same gene lists as the above clusterProfiler analysis [15]. Barcode plots from the GSEA analysis were plotted using the plotEnrichment() function in fgsea.

### Deeply sequenced subject—C9-FTD 4

To explore the effect of sequencing depth on CE detection a single subject was re-sequenced to a depth of 4,183,288,336 reads (252,629 reads per cell). The single subject was chosen for the high number of CEs detected and the inclusion of CEs in L5 extra telencephalic neurons. This subject had 13,079 cells across two libraries that were pooled equimolarly and sequenced on one lane of an S4 flowcell using the XP 4-Lane workflow using 101 × 12 × x12 × 101 cycles. Cellranger counts were used as above to align this sample to the human genome and generate a counts table for each 10x cell barcode in the sample. Regtools were run as described above to find *KALRN* and *STMN2* CE junctions on the annotated cell clusters. Nuclei barcodes containing a *KALRN* or *STMN2* CE were flagged in scanpy and highlighted on a UMAP. Differential expression analysis was then performed between nuclei that did or did not contain a *KALRN* or *STMN2* CE in the L2/L3 intratelencephalic neuron cluster with the covariate formula: ~ CE_YN + number_of_genes_in_the_cell. Pathway analysis using the GOBP database was then performed on this differentially expressed gene list using clusterProfiler. GSEA was performed using fsea on the pathway with the lowest qvalue or on a significant pathway of interest.

### Coverage analysis

Excitatory neurons (902) from subject C9-FTD 4 were analyzed for gene coverage in both regular and deeply sequenced BAM files. BAM files were generated by filtering the BAMs generated by cell ranger count from C9-FTD 4 to the same cells in both datasets. Coverage was determined for BAMs using bedtools coverage with the -d option to output coverage at each base. Coverage files were filtered to all exons in the ensembl primary (RefSeq; 34,791,404) isoform of each gene. Filtered coverage files were then parsed to determine the coverage at each base pair along each gene – going away from the 3´ end of the gene as measured from the terminal base in the final exon in the gene. Distance from the 3´ end was measured as the total exon distance, meaning distance was calculated using the full lengths of all exons in the primary transcript. Coverage at each base location was then averaged across all genes with any coverage in the data set. The coverage per gene was then divided by the number of included cells (902) to give the average coverage per gene per cell that is reported. Average coverage per gene per cell is plotted against the distance away from the 3´ end of the gene.

### IGV analysis

BAM files from our single-nuclei data were loaded into IGV to further investigate the presence of CE junctions. For sashimi plots, the junction tracks were filtered to only junctions with > 10 counts to focus on well-supposed junctions. This allows visual identification of the canonical exon-exon and novel exon-cryptic exon junctions in these datasets. To visually compare coverage depth within a gene region between samples, coverage (pile-up) track y-axis was scaled based on the relative number of genome-mapped reads in the sample.

### Data availability

The snRNA seq raw data are available at Synapse (syn45351388; 10.7303/syn45351388). Interactive data visualization and exploration of gene expression using CELLxGENE; https://cellxgene.cziscience.com/collections/aee9c366-f2fb-470b-8937-577d5d87d3fc.

## Results

**Fig. 1:**
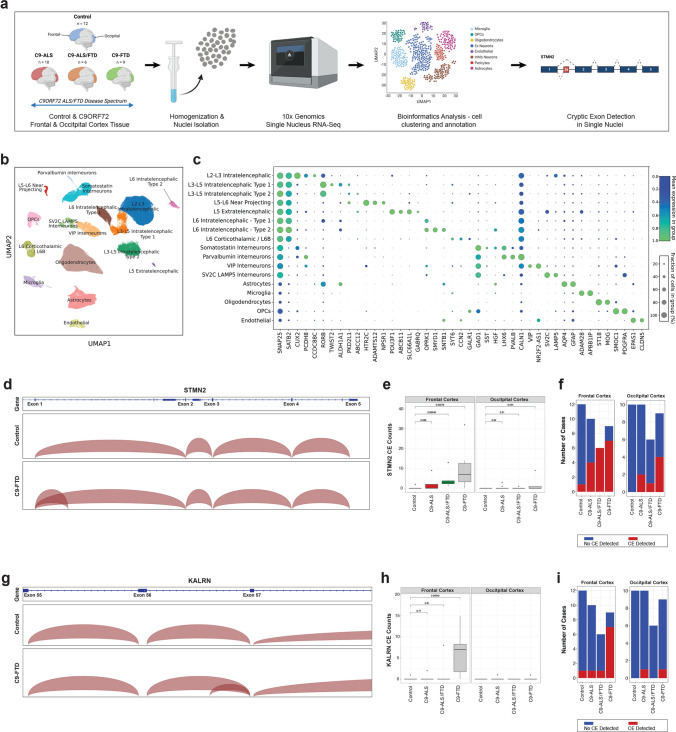
Detection of *STMN2* and *KALRN* cryptic exons in single-nuclei sequencing data from subjects with C9-ALS, C9-ALS-FTD, and C9-FTD.** a** Schematic diagram illustrating the workflow of nuclei isolation, single nuclei sequencing and analysis from the frontal and occipital cortices of subjects with C9-ALS (*n* = 10), C9-ALS-FTD (*n* = 6), and C9-FTD (*n* = 9), and aged-matched controls (*n* = 12). **b** UMAP depicting the 270,731 single nuclei sequenced from frontal cortex tissue separating into 17 distinct clusters. **c** Cell-type annotation performed based on the expression of previously described marker genes for each cell type in the frontal cortex. The size of the dot represents fraction of cells in which the marker gene was detected, and the color represents the average expression level in the cluster. **d** An IGV plot of the full-length *STMN2* gene. The top track shows data from the combined excitatory neuron clusters from control subjects (*n* = 12). The bottom track shows data from the combined excitatory neuron clusters from subjects with C9-FTD (*n* = 9). **e** Box plot of the average *STMN2* CE junctions detected per subject in each group. There is a significant increase in the detection of the *STMN2* CE between control subjects and C9-ALS/FTD (*p* = 0.00048; Wilcoxon test) and C9-FTD (*p* = 0.00079; Wilcoxon test) in the frontal cortex. There was also a significant increase in *STMN2* CE detection in the occipital cortex between control and C9-FTD subjects (*p* = 0.044, Wilcoxon test). **f**, Stacked bar plot displaying the number of subjects in which an *STMN2* CE was detected in the frontal cortex (left) and occipital cortex (right).** g** An IGV plot of the region of the *KALRN* gene containing the CE. The top track shows data from the combined excitatory neuron clusters from control subjects (*n* = 12). The bottom track shows data from the combined excitatory neuron clusters from patients with C9-FTD (*n* = 9). **h** Box plot of the average KARLN CE junctions detected per subject in each group. There is a significant increase in the detection of the *KALRN* CE in C9-FTD (*p* = 0.00042; Wilcoxon test) in the frontal cortex. **i**, Stacked bar plot displaying the number of individuals in which a *KALRN* CE was detected in the frontal cortex (left) and occipital cortex (right)

C9-ALS and C9-FTD exist on a disease spectrum sharing clinical and neuropathological similarities including progressive loss of neurons and TDP-43 pathology. To elucidate transcriptional differences, including TDP-43-associated CE inclusion, at a cell-specific level, we performed snRNA-seq on the frontal (disease-affected) and occipital (a less affected brain region) cortices of 12 neurologically normal controls and 25 *C9ORF72* patients whose clinical diagnosis spanned the ALS-FTD spectrum (C9-ALS *n* = 10, C9-ALS/FTD *n* = 6, C9-FTD *n* = 9) (Fig. 1a and Table 1; Supplementary Table 1, online resource). Following snRNA-seq and quality-control filtering, a total of 270,731 single nuclei were sampled from the frontal cortex, with a median of 3,509 genes and 9,218 transcripts detected per nucleus, and a total of 191,494 single nuclei were sampled from the occipital cortex with a median of 3,371 genes and 8,759 transcripts detected per nucleus. Visualization of the single-nuclei transcriptomes in uniform manifold approximation and projection (UMAP) space revealed unbiased separation of nuclei in the frontal cortex into 17 distinct clusters (Fig. 1b), with nuclei from both sexes and all disease types distributed across all clusters (Supplementary Fig. 1 a-d, online resource). Each cluster of nuclei in the frontal cortex was annotated on the basis of the expression of known cell-type-enriched markers for layer-specific and/or subtype-specific cortical neurons, in addition to non-neuronal cells (oligodendrocytes, oligodendrocyte progenitor cells (OPCs), astrocytes, microglia and endothelial cells) [3, 21, 32, 56] (Fig. 1b, c). The major neuronal and non-neuronal cell types were also identified in the occipital cortex by marker genes (Supplementary Fig. 1e, f, online resource). The number of cells varied within the tissue isolated; therefore, we provided the number of cells identified in each cell type across disease states, displayed in Supplementary Table 2 (online resource).

**Table 1:** Demographics of post-mortem samples used in this study

	Number of subjects	Sex	Age of onset (years ± SD)	Age at death (years ± SD)	Disease duration (years ± SD)
Control	12	6 F: 6 M	n/a	77.08 ± 11.96	n/a
C9-ALS	10	5 F: 5 M	53.22 ± 8.47	57.10 ± 8.46	2.59 ± 1.73
C9-ALS/FTD	6	3 F: 3 M	65.17 ± 7.49	67.17 ± 6.31	1.89 ± 0.98
C9-FTD	9	4 F: 5 M	58.56 ± 4.75	66.22 ± 5.05	7.67 ± 2.06

The identification of CE-containing cells could help identify cell populations vulnerable to TDP-43 pathology, thus we next sought to determine whether known CEs in transcriptional targets of TDP-43 could be detected in single cells within our snRNA-seq dataset. *STMN2* is currently the best-studied example of a gene in which TDP-43 dysfunction results in the inclusion of a CE between exon 1 and exon 2 in *STMN2* transcripts [2, 24, 39]. In our snRNA-seq dataset, we had read coverage of the *STMN2* gene in excitatory neurons across the full length of the *STMN2* transcript from the 5´ to the 3´ end of the gene, including the CE junction between exons 1 and 2 (Fig. 1d). As detection of the annotated or CE junction required the presence of a sequencing read crossing the junction, read coverage in this region enabled detection of the annotated exon 1 to exon 2 junction, as well as the CE junction (Fig. 1d). Figure 1d illustrates the detection of the annotated exon 1 to exon 2 junction in excitatory neurons in control subjects, and the addition of the CE junction between these exons in excitatory neurons isolated from C9-FTD subjects. Quantification of the number of *STMN2* CE junctions detected in the excitatory neurons in each of the disease groups showed that there was a significant increase in *STMN2* CEs in the C9-FTD group compared to controls in both the frontal cortex (*p* = 0.00079; Wilcoxon test) and the occipital cortex (*p* = 0.044; Wilcoxon test), as well as a significant increase in the C9-ALS/FTD group compared to controls in the frontal cortex (*p* = 0.00048; Wilcoxon test) (Fig. 1e). In total, the *STMN2* CE junction was detected in the frontal cortex of 7 of 9 subjects in the C9-FTD group, 6 of 6 subjects in the C9-ALS/FTD group, and 4 of 10 subjects in the C9-ALS group (Fig. 1f). The *STMN2* CE junction was also detected in 1 of 12 control subjects in the frontal cortex (Fig. 1f). Interestingly, *STMN2* CE junctions were also detected in the occipital cortex in all three of the *C9ORF72* disease groups, although in fewer subjects than in the frontal cortex (Fig. 1f). To our knowledge, this is the first-time single cells containing TDP-43-associated CEs have been detected and described in human postmortem brain tissue in a snRNA-seq dataset. Furthermore, given that the presence of CEs in known TDP-43 target transcripts is a potential indication of TDP-43 dysfunction [4, 23, 24, 29, 33, 39, 50], this is also the first transcriptomic description of single cells with TDP-43 dysfunction.

We next sought to detect CEs in other TDP-43 transcript targets. *KALRN* was recently demonstrated to include a CE in the absence of TDP-43 [33]. Using the bulk RNA sequencing dataset from Liu et al. (2019), which described the transcriptome of TDP-43 positive and negative nuclei from FTD-ALS postmortem brain with TDP-43 pathology [30], we confirmed the presence of a CE junction in the *KALRN* gene between exons 56 and 57, near the 3´ end of the gene (Supplementary Fig. 2b, online resource). This CE junction could also be detected in excitatory neurons in our snRNA-seq dataset. Figure 1g illustrates the detection of the canonical exon 56 to exon 57 junction in excitatory neurons in control subjects, and the addition of the CE junction between these exons in excitatory neurons derived from C9-FTD subjects (Fig. 1g). Similar to the *STMN2* CE, the number of *KALRN* CE junctions detected was significantly higher in the frontal cortex of C9-FTD subjects compared to controls (*p* = 0.00042; Wilcoxon test) (Fig. 1h). In total, the *KALRN* CE junction was detected in the frontal cortex of 7 of 9 subjects in the C9-FTD group, 1 of 6 subjects in the C9-ALS/FTD group, and 1 of 10 subjects in the C9-ALS group (Fig. 1i). It was also detected in 1 of 12 control subjects in the frontal cortex (Fig. 1i). In the occipital cortex the *KALRN* CE junction was not detected in any control or C9-ALS/FTD subjects and was only detected in 1 of 9 subjects in the C9-FTD group and 1 of 10 subjects in the C9-ALS group.

Another transcript described to include a CE upon depletion of TDP-43 in cellular models and in FTD-ALS postmortem tissue with TDP-43 pathology is *UNC13A* [4, 33]. We were unable to detect this CE junction in any nuclei in our snRNA-seq dataset (Supplementary Fig. 2a, online resource). Potential explanations for the lack of detection may include the lower average gene count for *UNC13A* compared with *STMN2* and *KALRN* in our dataset (Supplementary Fig. 2b, c, online resource), or low read coverage in the region of the *UNC13A* gene containing the CE (Supplementary Fig. 2a, online resource). Another possibility for not being able to detect the CE in *UNC13A* is the length of the CE from the 3´ end of the gene. The 10x Genomics sample preparation kits used for generating these data have a significant 3´ coverage bias because the priming for cDNA synthesis occurs at the poly(A) tail. We investigated how significant this bias was with respect to gene length. Supplementary Fig. 3a (online resource) displays the average read coverage, per cell, across the length of all genes identified. Files were filtered to include all exons, to make the longest possible isoform (using Ensembl; [6]), and then used as a reference to determine the coverage at each base pair starting from the 3´ end of the gene (where 10x Genomics priming starts), as measured by the end of the final exon in the gene. These data display higher coverage, as expected, at the 3´ end of the gene and low coverage towards the 5´ end. We then examined the largest list of potential CEs generated by the loss of TDP-43 function; 66 genes described by Ma et al. (2022) to be differentially spliced or to contain CEs in the TDP-43 negative neuronal nuclei obtained from Liu et al. (2019) dataset [30, 33]. Supplementary Table 3 (online resource) provides data on which of these genes we were able to detect a CE in the Liu et al. dataset and their chromosomal coordinates. We then looked for the detection of these CEs in our single nuclei data. For reference, the *UNC13A* CE is approximately 5627 base pairs (bp) from the 3´ end of the gene, using the longest possible isoform. This makes coverage over this region less likely with 10x Genomics RNASeq data. In contrast, the *STMN2* CE is approximately 227 bp from a premature polyadenylation site, making it within the peak of the 10x Genomics read depth. We looked at IGV plots for the three genes with the predicted closest CEs to the 3´ end; *TRAPPC12*, *MADD*, *RAP1GAP* (Supplementary Fig. 3b, d, f, online resource). There was very little read coverage in the region of the CEs for these genes, but this is possibly due to the low levels of expression of these genes (Supplementary Fig. 3c, e, g, h, online resource) compared with *STMN2* and *KALRN* (Supplementary Fig. 2b, c, online resource).

After identifying CEs in *STMN2* and *KALRN* by looking at the combined excitatory neurons, we then investigated whether a specific cell subtype was more likely to contain these CEs. The *STMN2* CE junction was detected in several excitatory and inhibitory neuronal subtypes, and non-neuronal cells across the *C9ORF72* disease groups with the highest abundance in L2–L3 intratelencephalic neurons in C9-FTD subjects (Table 2). The *KALRN* CE junction was almost exclusively detected in excitatory neuron subtypes, with no *KALRN* CE junctions detected in any inhibitory neuron subtype or non-neuronal cells in *C9ORF72* subjects, with the highest abundance in L2–L3 intratelencephalic neurons in C9-FTD subjects (Table 2). The differences in the cell type distribution of *STMN2* and *KALRN* CEs may be reflective of expression levels of the *STMN2* and *KALRN* genes in different cell types. *STMN2* gene expression is similar across all neuronal subtypes and lower in glial cells, while *KALRN* is expressed highest in excitatory neurons, lower in inhibitory neurons and astrocytes, and lower still in microglia and oligodendrocytes (Supplementary Fig. 2e, online resource). To determine which cell type was most vulnerable to CEs in *STMN2* and *KALRN*, we first normalized the number of CE junctions detected to the number of cells in each cluster (last three columns of Table 2). This revealed that there was a disproportionately high number of CE junctions detected in C9-FTD subjects in a very small cell cluster, L5 extratelencephalic neurons. Interestingly, the total cell counts for this neuronal subtype were found to be decreased in subjects with C9-FTD vs control subjects (cell numbers across groups are provided in Table 2; *p* = 0.043, Wilcoxon test).

**Table 2:** Detection of CEs for *STMN2* and *KALRN*, displayed as counts for each cell type and across each disease

	Total Cells				STMN2 CE Counts				KALRN CE Counts				CE Detected/ Cells in Cluster		
	Control (*n* = 13)	C9-ALS (*n* = 10)	C9-ALS-FTD (*n* = 6)	C9-FTD (*n* = 9)	Control	C9-ALS	C9-ALS-FTD	C9-FTD	Control	C9-ALS	C9-ALS-FTD	C9-FTD	C9-ALS	C9-ALS-FTD	C9-FTD
L2–L3 Intratelencephalic	11,132	23,300	9,763	11,619	**2**	**12**	**13**	**38**	0	**2**	**5**	**38**	0.0006	0.0018	0.0065
L3–L5 Intratelencephalic—Type 1	3952	3660	1888	2418	0	0	0	**2**	0	0	0	**2**	–	–	0.0017
L3–L5 Intratelencephalic—Type 2	4759	4337	2491	2844	0	0	**5**	**22**	0	0	**2**	**2**	–	0.0028	0.0084
L5-L6 Near Projecting	548	547	302	619	0	0	0	0	0	0	0	0	–	–	–
L5 Extratelencephalic	258	230	163	53	0	0	0	**6**	0	0	**1**	**2**	–	0.0061	0.1509
L6 Intratelencephalic—Type 1	3554	2414	1543	2124	0	**2**	**8**	**9**	**1**	0	0	**1**	0.0008	0.0052	0.0047
L6 Intratelencephalic—Type 2	873	671	366	736	0	0	0	0	0	0	0	**5**	–	–	0.0068
L6 Corticothalamic / L6B	2519	1879	944	1447	0	0	0	0	0	0	0	**1**	–	–	0.0007
Somatostatin Interneurons	3611	7505	2875	3436	0	0	**3**	0	0	0	0	0	–	0.0010	–
Parvalbumin interneurons	315	515	237	280	0	0	0	0	0	0	0	0	–	–	–
VIP Interneurons	2222	4750	1879	3103	0	0	0	**2**	0	0	0	0	–	–	0.0006
SV2C LAMP5 Interneurons	1098	1461	663	1748	0	0	0	0	0	0	0	0	–	–	–
Astrocytes	8048	8760	3184	10,565	0	**3**	0	**3**	**1**	0	0	0	0.0003	–	0.0003
Microglia	2328	2834	696	2631	0	0	0	**2**	0	0	0	0	–	–	0.0008
Oligodendrocytes	23,771	26,536	7702	22,291	0	0	**2**	**1**	0	0	0	0	–	0.0003	0.0000
Total	68,988	89,399	34,696	65,914	2	17	31	85	2	2	8	51			

Numbers were bolded to highlight the non-zero values in columns 6–13, as this represents cell types where cryptic exons were detected

**Fig. 2:**
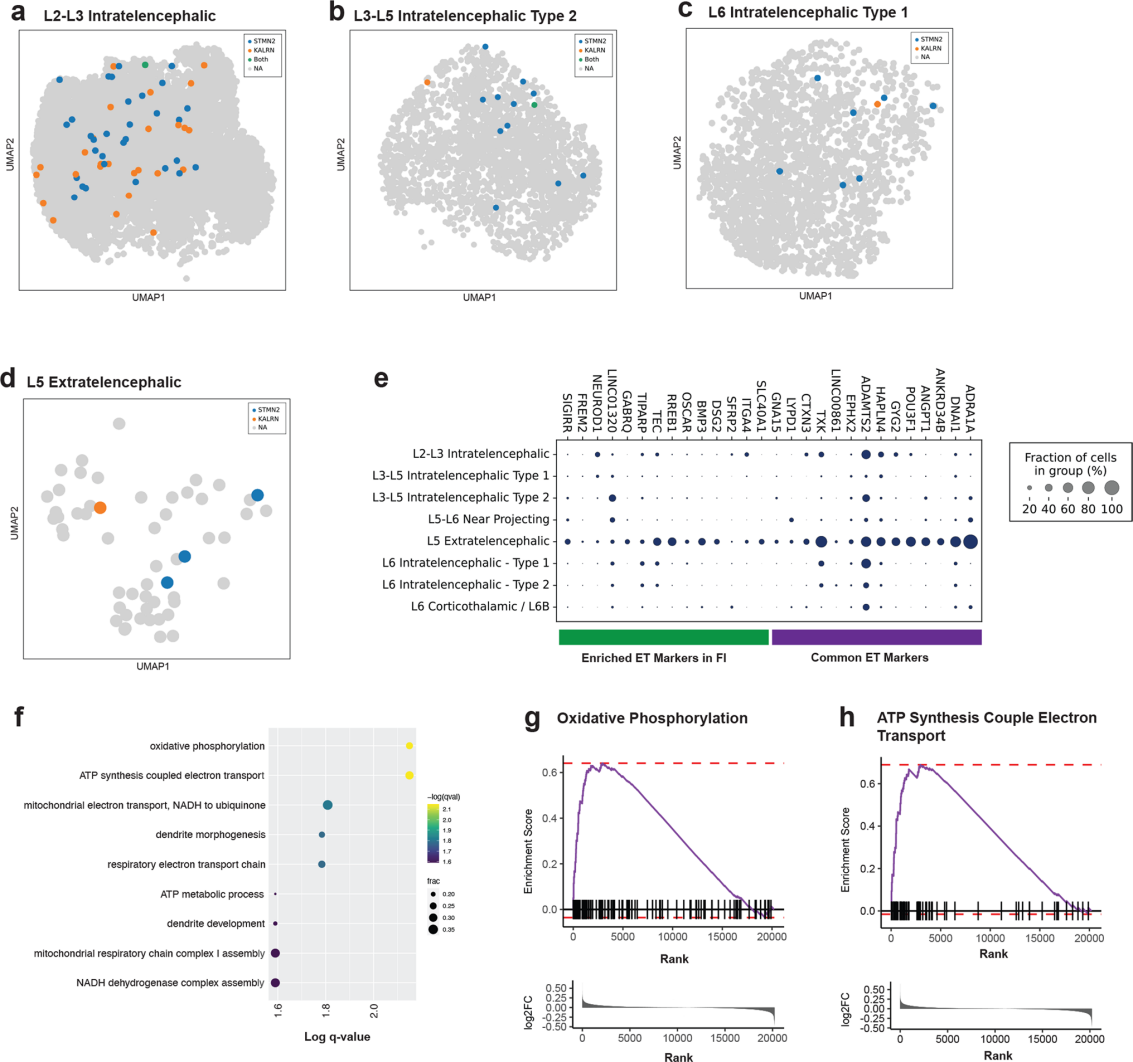
*STMN2* and *KALRN* CE detection in the four cell types with the highest number of CEs in C9-FTD. **a**–**d** UMAPs displaying the nuclei that contain a CE in *KALRN* (orange), *STMN2* (blue), or both *KALRN* and *STMN2* (green) laid over the non-CE containing nuclei (grey) in the **a**, L2–L3 intratelencephalic cluster, **b** the L3–L5 intratelencephalic type 2 neuron cluster, **c** the L6 intratelencephalic type 1 neuron cluster, and **d**, the L5 extratelencephalic neuron cluster. **e** Dot plot displaying the expression levels of Von Economo neurons marker genes taken from Hodge et al*.* in each excitatory neuron cluster [22]. The size of dot represents fraction of cells in which the marker gene was detected. **f** Pathway analysis using the differentially expressed genes between cells with detected CEs (*STMN2* and *KALRN* CE-containing cells combined) and cells with no CE detected in the L2–L3 intratelencephalic neuron cluster shown in (**a**). **g**–**h** Gene set enrichment data for the pathways with the lowest q value (0.0071) following clusterProfiler are displayed as barcode plots. **g** *Oxidative phosphorylation* and **h** *ATP Synthesis Couple Electron Transport*, showing an upregulation of gene expression; highest log_2_fc on the left to lowest on the right

As the presence of CEs in known TDP-43 target transcripts is thought to be an indication of TDP-43 dysfunction, we next investigated transcriptional changes occurring in cells containing the *STMN2* and *KALRN* CEs to provide insight into transcriptional effects of TDP-43 dysfunction. We first assessed whether *TARDBP* expression levels differed across cell types in control patient samples, and if this accounted for the vulnerability of certain cell populations to TDP-43 pathology and the incorporation of CEs. The mean expression of *TARDBP* in control nuclei was similar across all neuronal subtypes and large increases or decreases in expression were not associated with the neuronal subtypes that contained higher numbers of CE-containing transcripts (Supplementary Table 4, online resource). We next focused on the four cell clusters that had the highest number of CEs in C9-FTD: L2–L3 intratelencephalic neurons, L3–L5 intratelencephalic Type 2 neurons, L6 intratelencephalic Type 1 neurons and L5 extratelencephalic neurons. For each of these neuronal subtypes we identified every cell that contained a CE junction and visualized them in UMAP space (Fig. 2a–d). The L5 extratelencephalic neurons had the highest number of CEs detected in individuals with C9-FTD, relative to the size of the cell cluster, with 8 CEs detected in 53 neurons (0.1509 CEs detected per cell). Interestingly, we found that these cells express transcriptional marker genes that resemble Von Economo Neurons (VENs) and Fork cells based on a list of common extratelencephalic markers and markers found to be enriched in VENs generated by Hodge et al., who used snRNA-seq to characterize the transcriptome of VENs and Fork cells in Layer 5 of the human frontoinsular cortex (Fig. 2e) [22]. The L5 extratelencephalic neurons in our dataset display increased expression of these marker genes indicating that they have transcriptomic similarities to VENs and Fork cells of the frontoinsular cortex (Fig. 2e).

As the L2–L3 intratelencephalic neuron cluster contained the highest number of CE containing cells (*n* = 58), we performed pathway analysis on differentially expressed genes identified between the CE-containing cells and the non-CE-containing cells (Supplementary Table 4, online resource) of the cluster using clusterProfiler and the Gene Ontology Biological Process pathway database (Fig. 2f). The two pathways with the lowest q-value (*q* = 0.0071), ‘*Oxidative phosphorylation’* and ‘*ATP synthesis coupled electron transport’*, were assessed via Gene Set Enrichment Analysis (Fig. 2g, h). Both pathways display an increased expression in genes related to ATP and energy metabolism in the cells.

**Fig. 3:**
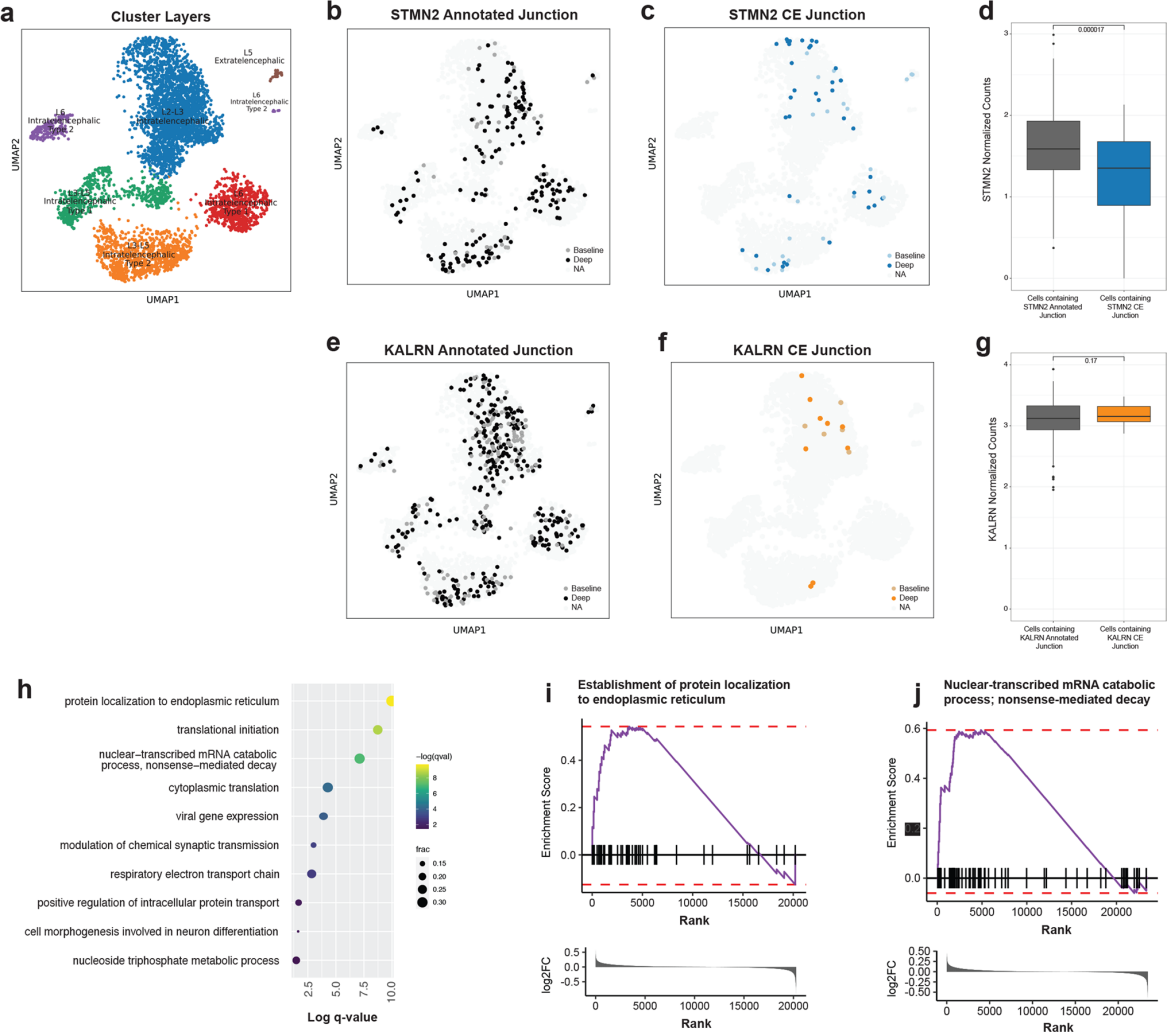
Deep sequencing reveals a larger number of *STMN2* and *KALRN* CE-containing nuclei. **a** UMAP displaying the 6 excitatory neuron clusters in which *STMN2* or *KALRN* CEs were detected. **b** Overlay on the UMAP in (**a**). Medium grey dots depict nuclei in which the annotated junction between exon 1 and exon 2 in *STMN2* was detected at the baseline level of sequencing, while additional cells containing an annotated junction identified after deeper sequencing are depicted in black circles. **c** An overlay of the nuclei in which an *STMN2* CE junction was detected in the baseline sequencing (light blue) and additional junctions detected with the deep sequencing (dark blue). **d** Box plot displaying normalized counts of STMN2 in the excitatory neurons containing an annotated STMN2 junction compared with excitatory neurons containing an STMN2 CE junction in subject C9-FTD 4 (*p* = 0.000017, *t*-test). **e** Overlay on the UMAP in (**a**). Medium grey dots depict nuclei in which the annotated junction between exon 56 and exon 57 in *KALRN* was detected at the baseline level of sequencing, while additional cells containing an annotated junction identified after deeper sequencing are depicted in black circles. **f** An overlay of the nuclei in which a *KALRN* CE junction was detected in the baseline sequencing (light orange) and additional junctions detected with the deep sequencing (dark orange). **g** Box plot displaying normalized counts of *KALRN* in excitatory neurons containing an annotated *KALRN* junction compared with excitatory neurons containing a *KALRN* CE junction in subject C9-FTD 4 (*p* = 0.17, *t*-test). **h** Pathway analysis using the differentially expressed genes between cells with detected CEs in deeply sequenced data (*STMN2* and *KALRN* CE-containing cells combined) and cells with no CE detected. **i**, Barcode plot of gene set enrichment analysis displays upregulation of gene expression in the pathway with the lowest q value (*q* = 1.24e–04) (**f**)*, establishment of protein localization to endoplasmic reticulum*. **j** Gene set enrichment analysis displays upregulation of gene expression in the significant (*q* = 0.008) pathway *nuclear-transcribed mRNA catabolic process*; *nonsense-mediated decay*

The detection of CE junctions in our snRNA-seq data is likely an underestimation due to the sparse nature of the 10x Genomics technology used to sequence the data, as there is less read depth and shorter read lengths than is typically acquired with bulk RNA sequencing. We, therefore, evaluated whether increasing the read depth would increase the number of cells with CE junctions detected and increase the total number of detected junctions per cell. We chose to deeply sequence one subject (C9-FTD 4) in our data because this subject had the largest number of *STMN2* CE junctions in the cell cluster with the highest proportion of CEs, the L5 extratelencephalic neurons. Following deeper sequencing, the number of reads in the C9-FTD 4 sample increased from 519,837,269 to a total of 4,183,288,336 reads, and the average number of reads per cell increased from 36,897 to 252,629. The number of CE junctions detected in both *STMN2* and *KALRN* increased by approximately eightfold following deeper sequencing, with the number of *STMN2* CE detected increasing from 33 to 252, and *KALRN* CE detection increasing from 6 to 59 (Table 3). In the L5-extratelencephalic cluster, the number of CEs detected per the number of cells in the cluster increased from 0.16 to 0.65. To visualize the impact of deeper sequencing on the number of cells with a detectable CE junction, we displayed all the excitatory neuron clusters that had a detectable CE in UMAP space, 5202 cells separated into 6 clusters (Fig. 3a). We identified the cells where the annotated exon junction and the CE junctions were detected for both *STMN2* (Fig. 3b, c) and *KALRN* (Fig. 3e, f). For *STMN2* prior to deep sequencing, the annotated exon 1 to exon 2 junction was detected in 46 cells (Fig. 3b) and the CE junction was detected in 21 cells (Fig. 3c). Following deep sequencing, these numbers increased to 213 cells containing the annotated exon 1 to exon 2 junction and 58 cells containing the CE junction. Following baseline sequencing, the annotated exon 56 to exon 57 junction in *KALRN* was detected in 159 cells, which increased to 438 cells after deeper sequencing (Fig. 3e). However, detection of the *KALRN* CE junction only increased from 5 to 14 cells following deeper sequencing (Fig. 3f). For *KALRN*, deeper sequencing had less of an impact on the detection of CE junctions, possibly because *KALRN* is already more highly expressed in these cells (Supplementary Fig. 2d, e, online resource). We also assessed whether the inclusion of a CE, a potential indicator of TDP-43 pathology, affected the expression level of each of these genes. The mean expression of *STMN2* was significantly reduced in deeply sequenced cells containing *STMN2* transcripts with the inclusion of the CE, compared to deeply sequenced cells containing *STMN2* transcripts with the annotated junction (*p* = 0.000017; t-test) (Fig. 3d, and using all neurons in Supplementary Fig. 4a, online resource). This pattern of reduced *STMN2* expression in cells containing CE-containing transcripts was also observed using the Liu et al. (2019) dataset, as TDP-43 negative nuclei (CE-containing nuclei) had a significantly lower expression of *STMN2* than TDP-43 positive nuclei (*p* = 0.0028; t-test) (Supplementary Fig. 4c, online resource). In contrast to *STMN2* expression, there was no significant difference in the mean expression of *KALRN* in cells containing *KALRN* transcripts with the inclusion of the CE, compared to cells containing *KALRN* transcripts with the annotated junction (*p* = 0.17; t-test) (Fig. 3g, and using all neurons in Supplementary Fig. 4b, online resource). This differed from the Liu et al. (2019) dataset, where TDP-43 negative nuclei (CE-containing nuclei) had a significantly higher mean expression of *KALRN* compared to TDP-43 positive nuclei (*p* = 0.0088; t-test) (Supplementary Fig. 4d, online resource).

**Table 3:** Cryptic exon detection for *STMN2* and *KALRN* in a deeply sequenced subject (C9-FTD 4)

Subject: C9-FTD 4	Number of cells sequenced	Baseline Sequencing			Deep Sequencing		
		STMN2 CE junctions	KALRN CE junctions	CE detected/ Cells in cluster	STMN2 CE junctions	KALRN CE junctions	CE detected/ Cells in cluster
L2–L3 Intratelencephalic	2,830	8	6	0.005	83	53	0.048
L3–L5 Intratelencephalic—Type 1	582	1	0	0.002	10	0	0.017
L3–L5 Intratelencephalic—Type 2	901	13	0	0.014	93	6	0.110
L5-L6 Near Projecting	169	0	0	0.000	0	0	0.000
L5 Extratelencephalic	37	6	0	0.162	24	0	0.649
L6 Intratelencephalic—Type 1	674	4	0	0.006	36	0	0.053
L6 Intratelencephalic—Type 2	178	0	0	0.000	0	0	0.000
L6 Corticothalamic/L6B	315	0	0	0.000	0	0	0.000
Somatostatin Interneurons	876	0	0	0.000	0	0	0.000
Parvalbumin interneurons	94	0	0	0.000	0	0	0.000
VIP Interneurons	498	0	0	0.000	0	0	0.000
SV2C LAMP5 Interneurons	276	0	0	0.000	0	0	0.000
Astrocytes	1644	1	0	0.001	6	0	0.004
Microglia	267	0	0	0.000	0	0	0.000
Oligodendrocytes	3738	0	0	0.000	0	0	0.000
Total	13,079	33	6	0.003	252	59	0.024

As deeper sequencing increased the number of cells presumed to be affected by TDP-43 dysfunction due to the presence of *STMN2* and/or *KALRN* CE junctions, we again performed differential gene expression analysis on the largest neuronal subgroup (L2–L3 intratelencephalic neurons) between nuclei containing a CE (either *STMN2* and/or *KALRN*) and nuclei not containing a CE for the deeply sequenced subject; C9-FTD 4 (Supplementary Table 5, online resource). Pathway analysis of the DEGs found the most significantly affected biological pathway impacted in CE-containing cells to be ‘*protein localization to the endoplasmic reticulum’* (*q* = 1.24e-04; Fig. 3h). We also examined the significant ‘*nonsense mediated decay pathway’* (*q* = 0.008) (Fig. 3h). These two pathways were assessed with Gene Set Enrichment Analysis and barcode plots to indicate the direction of expression of the genes (Fig. 3i, j). Both pathways showed an upregulation of gene expression in CE containing cells.

## Discussion

The GGGGCC (G_4_C_2_) hexanucleotide repeat expansion in the first intron of the *C9ORF72* gene is the most common genetic abnormality associated with ALS and FTD [7, 52]. While TDP-43 pathology is one of the hallmarks of *C9ORF72* disease, the molecular and cellular mechanisms underlying cortical neurodegeneration in this disease remain largely understudied, and the cell-type specific impacts of TDP-43 pathology are unknown. Here, we performed snRNA-seq analyses of frontal and occipital cortices from all subgroups of *C9ORF72* patients with the goal of providing a comprehensive, cell-type specific transcriptomic profile across the *C9ORF72* spectrum. In addition, using this dataset, we show for the first time the detection of CEs in single nuclei for two known splicing targets of TDP-43 and, using this as a hallmark of TDP-43 dysregulation, we assessed gene expression changes in these cells to provide insight into the transcriptome of individual cells with TDP-43 pathology.

Several recent studies indicate that nuclear depletion of TDP-43 leads to dysregulation in pre-mRNA splicing and results in a failure of TDP-43 repressing the inclusion of intronic sequences that become incorporated into mature mRNA as CEs. Detection of CEs in a specific transcriptional target of TDP-43, such as *STMN2*, is considered a hallmark of TDP-43 dysregulation and a potential way to identify cells with TDP-43 nuclear depletion [4, 24, 29, 33, 39, 50]. Initially, we focused on the detection of a previously described CE in *STMN2*. We also detected a recently identified CE in *KALRN*. The *STMN2* CE is detected at higher rates in excitatory neurons than *KALRN* in these data, despite *STMN2* being expressed at lower levels. The number of both these CEs is significantly higher in *C9ORF72* tissues compared to controls. For *STMN2*, the number of CEs detected in each *C9ORF72* subgroup reflected the expected level of TDP-43 pathological burden in the frontal cortex, with C9-FTD tissues having the highest *STMN2* CE counts and C9-ALS tissues having the lowest. In contrast, the *KALRN* CE was more specific to the C9-FTD tissue. A possible explanation for this could be dose-dependent effects of TDP-43 dysfunction on CE inclusion, as has been suggested for *STMN2* and *UNC13A* [4, 39]. It is possible that *STMN2* requires a lower level of TDP-43 dysfunction for the CE to be included, while the inclusion of the CE in *KALRN* may require higher losses of TDP-43 function, although this hypothesis needs further testing. An alternative hypothesis is that different CE-containing transcripts may be vulnerable in the different phenotypic presentations of the *C9ORF72* mutation, with *KALRN* CEs being more specific to an FTD presentation of disease, although the *KALRN* CE was recently identified in an analysis of ALS patient postmortem spinal cord [63], thus further investigation is required to explore this hypothesis. It is also interesting to note that CEs for *STMN2* were found in seven occipital cortices having the *C9ORF72* repeat expansion, and the *KALRN* CE was detected in two *C9ORF72* cases in the occipital cortex, indicating that this brain region may not be unaffected by TDP-43 dysfunction. A careful examination of TDP-43 pathology and changes in the occipital cortex transcriptome should be performed in future studies.

After identifying CEs in *STMN2* and *KALRN* in the combined excitatory neuron clusters, we investigated whether there were subtypes of excitatory neurons that were more susceptible to CEs in *STMN2* and *KALRN*. Neuronal subtypes of the frontal cortex were annotated (Fig. 1c) using the expression of marker genes previously identified in a snRNA-seq study of the human frontal cortex [32]. Although the cluster identified as L2–L3 intratelencephalic neurons contained the highest number of *STMN2* and *KALRN* CEs (Table 2), the cluster with the greatest proportion of cells with CEs was the L5 extratelencephalic neuronal cluster. Despite being the smallest neuronal cell cluster identified in our dataset, the high proportion of cells containing a CE in this cluster may suggest that this neuronal subtype is vulnerable to TDP-43 dysfunction. In addition, the number of L5 extratelencephalic neurons identified in the frontal cortex of C9-FTD subjects was significantly lower than in controls, further supporting the idea that these cells may be vulnerable in disease. Further investigation into this neuronal subtype revealed that the L5 extratelencephalic excitatory neuron cluster has a similar transcriptional profile to VENs and Fork cells. Marker genes identified by Hodge et al*.,* in a single-nuclei RNA sequencing analysis of layer 5 of the human frontoinsular cortex [22], described well-characterized VEN markers including, *GABRQ*, *ADRA1A*, and *LYPD1* that were also enriched in the L5 extratelencephalic neurons [8, 12, 59]. VENs are believed to be restricted to the anterior cingulate and frontoinsular cortex in humans [18, 53]; however, there are reports of VENs being present on the medial surface of the superior frontal gyrus (Brodmann Area 9) and polar region of medial Brodmann Area 10, albeit to a lesser extent than in the anterior cingulate [10, 17]. VENs and fork cells in *C9ORF72*-FTD cases are more likely to have TDP-43 aggregation and TDP-43 nuclear depletion than neighboring layer 5 neurons [44]. The L5-L6 near projecting neuronal cluster had no detectable *STMN2* or *KALRN* CEs (Table 2), thus it is possible that this cell type may be spared of TDP-43 dysfunction, or CEs in other transcripts not assessed in this study may be more prevalent in this cell type. Further studies are required to understand why some neuronal subtypes are more vulnerable than others to *STMN2* or *KALRN* CEs, and by extension, TDP-43 dysfunction.

With regard to other cell types, a low number of *STMN2* or *KALRN* CE-containing transcripts were detected in interneurons and glial cells. The lower detection in the glial cells is likely related to the higher expression of *STMN2* and *KALRN* in neurons compared to glial cells (Supplementary Fig. 2c, online resource). Furthermore, it should be noted that most studies focused on identifying CEs in TDP-43 transcriptional targets have done so in neurons or neuronal cell-types, which may have resulted in a bias for transcriptional targets that are more specific to neurons [4, 24, 30, 33, 39, 50]. It is, therefore, possible that glial cells have different transcriptional targets of TDP-43 that harbor CEs in gene transcripts more relevant to these cell types.

Identifying specific cells that contained either an *STMN2* or *KALRN* CE allowed us to explore the transcriptomic changes of those cell types in which we detect a CE and are likely affected by TDP-43 pathology. To obtain such a TDP-43 pathology-associated, cell-type specific transcriptomic signature, we focused on the L2–L3 intratelencephalic neuron cluster as this cluster contained the most cells containing CEs. Differential gene expression and subsequent pathway analysis revealed an increased expression in genes related to oxidative phosphorylation, ATP synthesis and energy metabolism in the cells containing either *STMN2* or *KALRN* CEs. This indicates cells containing CE-containing transcripts that are likely associated with TDP-43 pathology have altered energy metabolism demands, which is a common feature linked to several neurodegenerative diseases [14, 41]. This finding also supports a previous study that reported destabilization of RNAs encoding oxidative phosphorylation and ribosome components in patient-derived C9-ALS cell models, in control induced pluripotent cells overexpressing TDP-43, and ALS and FTD postmortem brain and spinal cord [55]. Together these findings implicate abnormalities in the oxidative phosphorylation and ribosomal pathways in ALS and FTD characterized by TDP-43 pathology.

Differential gene expression and pathway analysis performed on L2–L3 intratelencephalic neuron cluster in the deeply sequenced subject also implicated protein localization to the endoplasmic reticulum and NMD as two additional pathways that are upregulated in cells containing *STMN2* or *KALRN* CEs. Given the findings for changes in protein localization in the ER and the changes in mitochondrial function found in Fig. 2f, it is possible that ER-mitochondrial signaling is impaired in CE-containing cells. ER-mitochondrial signaling has been found to be disrupted in many neurodegenerative diseases, including FTD and ALS (reviewed in [25]). One important feature of ER-mitochondrial signaling is to bring the organelles into close proximity with tethering proteins, which have been shown to be disrupted in FTD and Alzheimer’s disease [25, 26, 38]. Disruptions in organelle signaling and proximity between the ER and mitochondria can lead to changes in ATP production, and mitochondrial function (Fig. 2f), as well as synaptic damage [16, 20] (Figs. 2f and 3h). This will be important to investigate in future studies. The dysregulation of ‘*nonsense mediated decay pathway’* in CE-containing cells is interesting given that it has been suggested that many transcripts containing CEs are degraded by the process of NMD, as is hypothesized to happen with UNC13A [4, 9, 23, 29, 33, 54]. Furthermore, alternative splicing coupled to NMD (AS-NMD) is an important post-transcriptional mechanism for regulating gene expression and is known to be involved in maintaining homeostatic expression and autoregulation of many RNA-binding proteins [13, 27, 42, 49, 62]. The reason for the upregulation of the NMD pathway is unclear from these data. It is possible that NMD-associated genes are upregulated in response to CE-containing transcripts as a mechanism to clear these transcripts from the cell. Alternatively, impairment of the AS-NMD process may be contributing to the inclusion of the CEs in transcripts, as the expression of many transcripts is regulated by this post-transcriptional mechanism. Given the well-described dysfunction of many RNA-binding proteins in ALS and FTD, including TDP-43, the upregulation of NMD and its roles as both a surveillance mechanism and homeostatic regulator should be explored further in the context of TDP-43 pathology and CE inclusion in disease.

We also observed that the mean expression level of *STMN2* was reduced in CE-containing cells compared to cells containing the annotated junction (Fig. 3d and Supplementary Fig. 4a). This is in accordance with previous reports of reduced *STMN2* mRNA expression in TDP-43 knock-down models that result in the production of the CE-containing transcript [2, 24, 39]. In contrast, *KALRN* expression was not significantly altered in *KALRN* CE-containing cells in our snRNA-seq dataset, but analysis of the Liu et al. (2019) dataset indicated a significant increase in *KALRN* expression in TDP-43 negative cells. It is interesting that the inclusion of a CE has different consequences for the expression of *STMN2* and *KALRN*, which may point to the CEs having different functional impacts on these two transcripts and should be explored further.

There are limitations to the interpretation of these data that should be addressed. As stated previously, one of the obvious caveats of 10x Genomics data is the strong 3´end bias and sparse transcript coverage in the data (Supplementary Fig. 3, online resource). For example, a CE in the *UNC13A* gene has been associated with TDP-43 dysfunction [4, 33]. We were unable to detect this CE, or other CEs reported in other genes in our snRNA-seq data set, despite the *UNC13A* gene being detectable in the frontal cortex of the tissues analyzed (Supplementary Fig. 2a, online resource). Technical issues associated with 10x Genomics single-nuclei sequencing resulted in low read coverage in the region of the *UNC13A* CE. Low read coverage can be due to low expression of the gene in individual cells (both *STMN2*, and especially *KALRN*, had higher gene expression than *UNC13A*), or the sparse nature of single nuclei data (low read depth within individual cells). The 3 additional genes that we displayed more fully in Supplementary Fig. 3, *TRAPPC12*, *MADD,* and *RAP1GAP*, had CEs predicted to be close to the 3´end, and these 3 genes had very low expression compared to *KALRN* (Supplementary Fig. 2b and 3h). The 3´end bias also likely impacts the lack of detection of the *UNC13A* CE, and other reported CEs because these CEs are located further from the 3´ end of the gene. It is also important to note that the sequencing of nuclei, rather than cells, may hinder the ability to detect CEs because many of the transcript’s sequenced are likely pre-spliced mRNAs and thus neither have an annotated exon junction nor CE.

Another important factor to consider when interpreting this data is the possibility of cells containing undetected CEs. As stated previously, in any of the analyses, we only detect a small number of reads that span the junctions of interest in this study. In the deeply sequenced sample, we detect a read crossing the annotated junctions for *STMN2* (exon 1–2, approximately 1805 bp from 3´ end) in 213 cells of 5202 (4%) and we detect the annotated *KALRN* junction between exons 56–57) in 438 cells of 5202 (8%). It is highly likely that the number of cells containing the annotated junctions is much higher, and our data therefore likely underestimates both the number of cells containing annotated junctions and CE junctions. This underestimation may mean that some cells labeled as non-CE-containing nuclei may contain an undetected CE. This caveat is important when considering the differentially expressed genes and pathway analysis performed in this study.

Additional limitations of this study are that we did not perform discovery for CEs in different cell clusters, and therefore, may have potentially missed CEs that are important and unique to each cell type, particularly glial cells. In addition, there are a small number of subjects used in these comparisons and the addition of more subjects in the validation of these findings would be advantageous. Furthermore, the variability of the precise sample location from tissue could have an impact on the number of cell types detected from each subject, and the potential to include rare cell types, such as L5 extratelencephalic cells.

In summary, we show for the first time the presence of CEs in known transcriptional targets of TDP-43 in select, neuronal subtypes and disease-associated brain regions using single-nuclei RNA-sequencing technology. This allowed us to assess the cell-type-specific transcriptomic changes of presumed cells affected by TDP-43 pathology within an endogenous disease context. This is particularly important as limited information on cell-type specific vulnerability to TDP-43 pathology in the frontal cortex of *C9ORF72* patients has been available. These data provide a first insight into the disruption of cortical neuronal subtypes caused by the inclusion of CEs as a downstream consequence of pathologic TDP-43 nuclear depletion. Further investigations should consider the use of long-read sequencing technologies in combination with single-nuclei/single-cell separation to provide a more comprehensive quantification and description of CE-containing cells.

## Supplementary Information

Below is the link to the electronic supplementary material.

Supplementary file1 (XLSX 5457 kb)

Supplementary Table 1. Additional case demographics for the cohort used in this study

Supplementary Table 2. Number of nuclei detected in each cell type in each case used in this study

Supplementary Table 3. Genes previously identified to be alternatively spliced or have cryptic exons in TDP-43 negative nuclei

Supplementary Table 4. Mean *TARDBP* expression in neuronal clusters

Supplementary Table 5. Differentially expressed genes between CE-containing cells and cells in which CE were not detected in each excitatory neuronal subtype

Supplementary Table 6. Differentially expressed genes between CE-containing cells and non-CE-containing L2–L3 intratelencephalic cells in subject C9-FTD 4
