# Supplementary material for: Cryptic exon detection and transcriptomic changes revealed in single-nuclei RNA sequencing of C9ORF72 patients spanning the ALS-FTD spectrum
Source: Acta Neuropathol. 2023 Jul 19;146(3):433–50. doi: 10.1007/s00401-023-02599-5 (PMC10412668; doi:10.1007/s00401-023-02599-5)
Supplement: Supplementary file 7 — Supplementary Table 6. Differentially expressed genes between CE-containing cells and non-CE-containing L2–L3 intratelencephalic cells in subject C9-FTD 4 [file 401_2023_2599_MOESM7_ESM.pdf]

Supplement Figure 1.

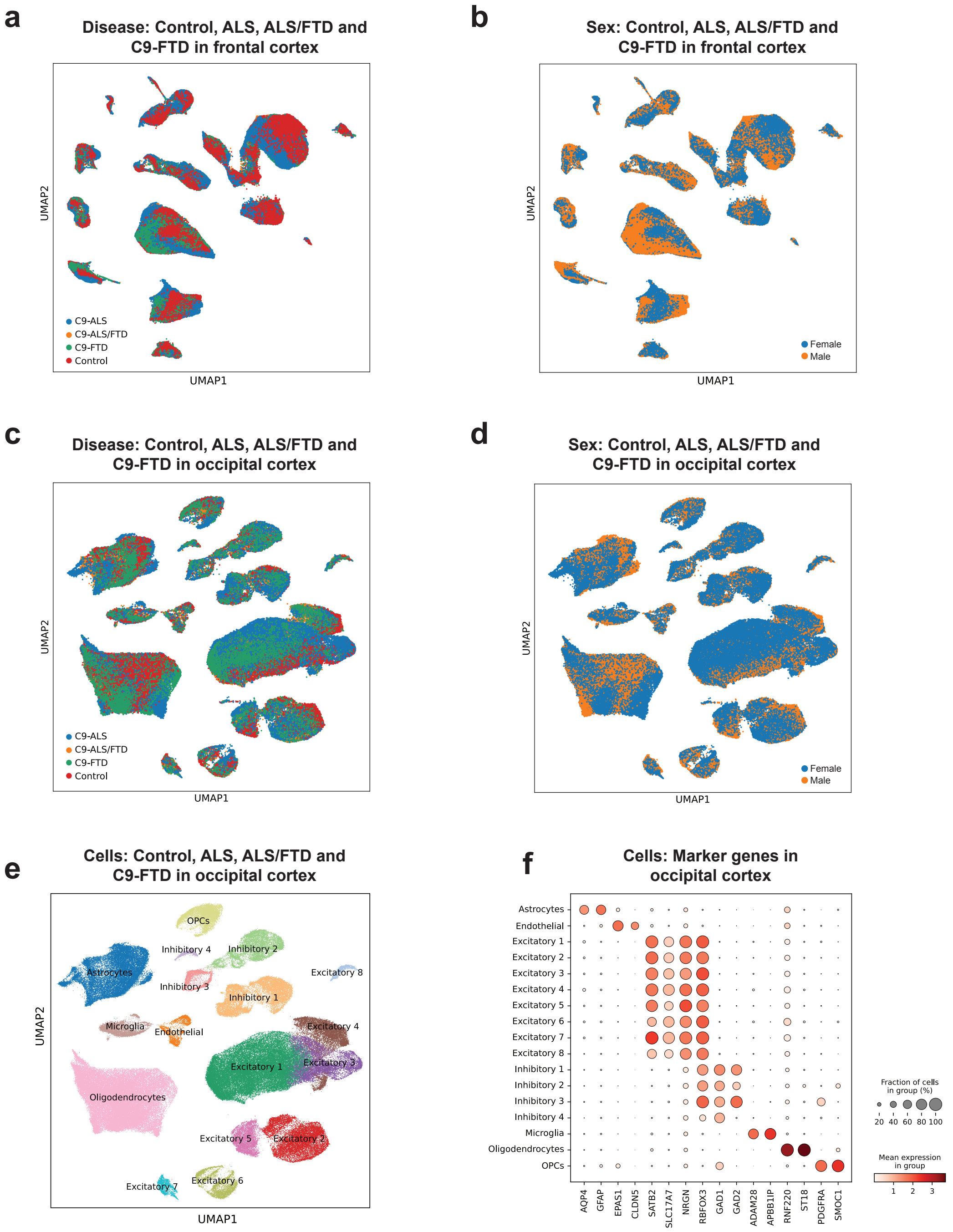

UMAPs for disease and sex in the frontal and occipital cortices. a, UMAP depicting nuclei from each of the diseases (*n* = 12 control; *n* = 10 C9-ALS; *n* = 6 C9-ALS-FTD; and *n* = 9 C9-FTD) in the frontal cortex separated into 17 cell clusters. **b**, UMAP depicting nuclei from male (*n* = 19) and female subjects (*n* = 18) in the frontal cortex. **c** UMAP depicting nuclei from each of the diseases *(n* = 10 control; *n* = 10 C9-ALS; *n* = 6 C9-ALS-FTD; *n* = 9 C9-FTD) in the occipital cortex separated into 17 cell clusters **d**, UMAP depicting nuclei from male (*n* = 19) and female subjects (*n* = 16) in the occipital cortex. **e**, UMAPs depicting the major cell types in the occipital cortex. **f**, Cell-type annotation performed based on previously described marker genes for each cell type in the occipital cortex. The size of dot represents fraction of cells in which the marker gene was detected, and the intensity of the color represents mean expression level in group.

Supplement Figure 2.

a

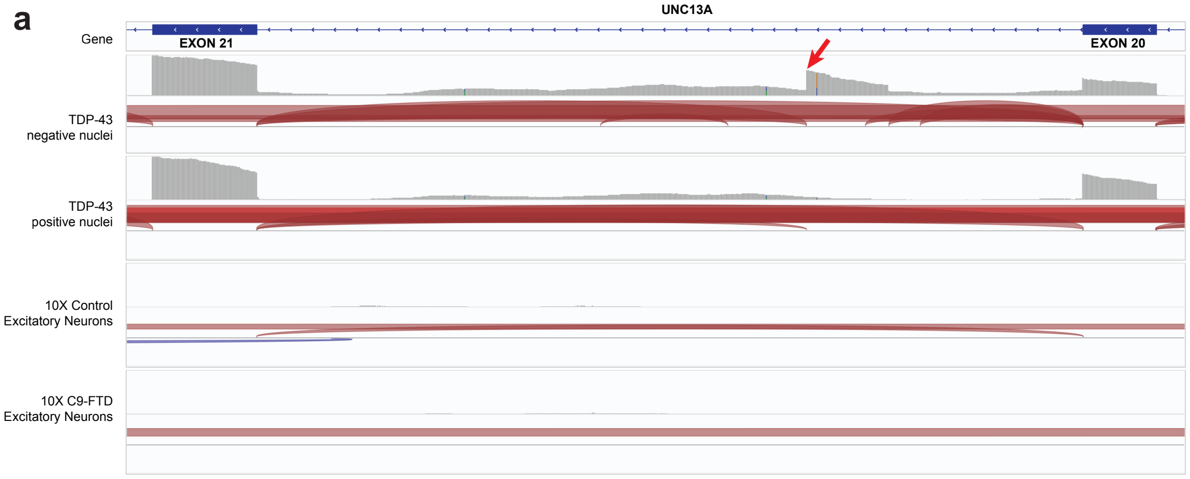

b

	Control	C9-ALS	C9-ALS/FTD	C9-FTD
**STMN2**	1.18	1.51	1.46	1.12
**KALRN**	3.27	3.13	3.21	3.31
**UNC13A**	1.10	1.11	0.99	1.03

c

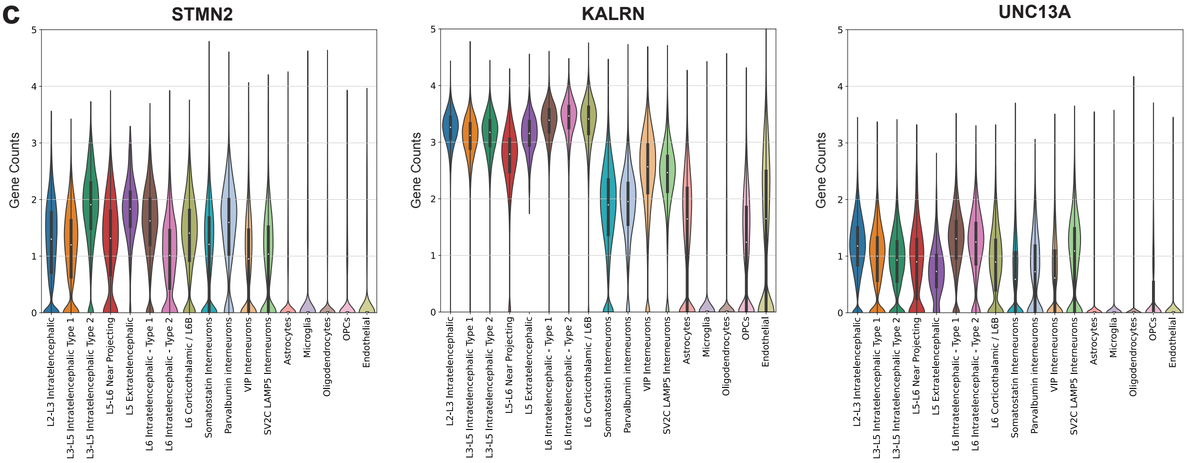

The *UNC13A* CE is not detected in frontal cortex excitatory neurons in our 10x Genomics snRNA-sequencing dataset.

a, IGV plot displaying the region of the CE in *UNC13A*. The top track shows the detectable UNC13A CE in TDP-43-/NeuN+ nuclei from Liu *et al.* (2019). The arrow points to the position of the CE junction. The second track is the same region in the TDP-43+/NeuN+ nuclei from Liu *et al.* (2019). The third track is the excitatory neurons from the control samples (n = 12) in our 10x Genomics data. The fourth track is the excitatory neurons from C9-FTD subjects (n = 9). **b,** A table displaying the mean expression levels of *STMN2*, *KALRN*, and *UNC13A* in Controls, C9-ALS, C9-ALS/FTD and C9-FTD in all excitatory neurons in our snRNA sequencing dataset **c**, Violin plots showing the expression levels of *STMN2*, *KALRN*, and *UNC13A* in each of the cell clusters in the frontal cortex.

Supplement Figure 3.

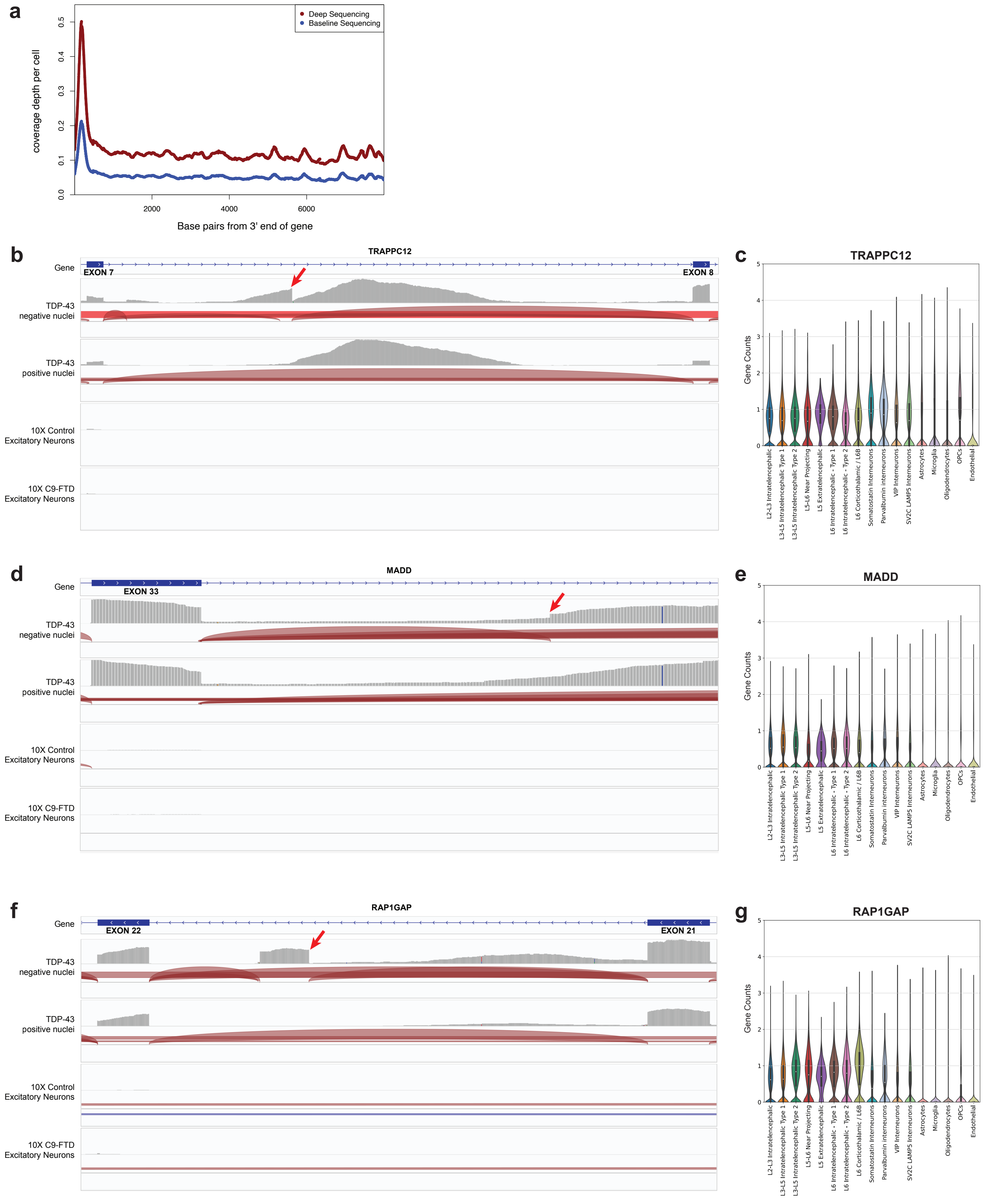

h

	Control	C9-ALS	C9-ALS/FTD	C9-FTD
TRAPPC12	0.76	0.70	0.67	0.69
MADD	0.53	0.55	0.44	0.44
RAP1GAP	0.72	0.68	0.63	0.61

Supplementary Fig. 3

CEs in TRAPP12C, MADD and RAP1GAP are not detected in frontal cortex excitatory neurons in C9-FTD our 10x Genomics snRNA-sequencing dataset.

a, Plot displaying the 10x Genomics data coverage of transcripts from the 3' to the 5' end. 10x Genomics data has a significant 3' bias due to technical aspects of their sequencing protocol. This plot uses excitatory neurons from C9-FTD 4 (n = 902 cells) and is calculated as the average coverage at each base starting at the 3' end of the gene. The baseline (blue) sequencing and the deep (maroon) sequencing for the same 902 cells are displayed. **b**, **d**, **f**, IGV plots for the region of the CEs in *TRAPPC12* (**b**), *MADD* (**c**), and *RAP1GAP* (**d**). In each of the IGV plots the top track shows the TDP-43-/NeuN+ nuclei from Liu *et al.* (2019). The red arrows point to the position of the CE junction. The second track is the same region in the TDP-43+/NeuN+ nuclei from Liu *et al.* (2019). The third track is the excitatory neurons from the control samples (n = 12) in our 10x Genomics data. The fourth track is the excitatory neurons from C9-FTD subjects (n = 9). **c**, **e**, **g**, Violin plots showing the expression levels of *TRAPPC12* (**c**), *MADD* (**e**), and *RAP1GAP* (**g**) in each of the cell clusters in the frontal cortex. **h**, A table displaying the mean expression levels of *TRAPP12*, *MADD*, and *RAP1GAP* in excitatory neurons from Controls, C9-ALS, C9-ALS/FTD and C9-FTD in our snRNA sequencing dataset.

Supplement Figure 4.

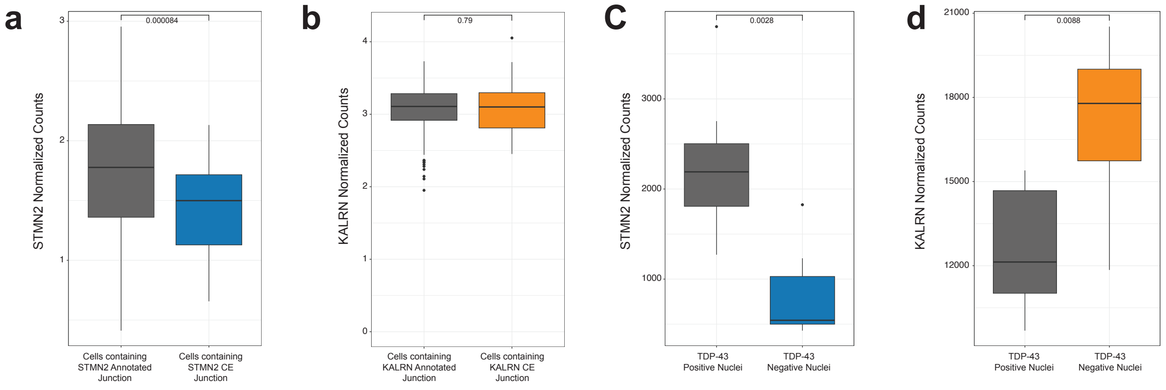

The effect of CE inclusion and TDP-43 depletion on the expression of *STMN2* and *KALRN.*

a, Box plot displaying normalized counts of *STMN2* in excitatory neurons containing an annotated *STMN2* junction compared with excitatory neurons containing a *STMN2* CE junction from all C9-FTD individuals (*p* = 0.000084, t-test). **b**, Box plot displaying normalized counts of *KALRN* in excitatory neurons containing an annotated *KALRN* junction compared with excitatory neurons containing a *KALRN* CE junction (*p* = 0.79, t-test). **c**, Box plot displaying normalized counts of *STMN2* in TDP-43+/NeuN+ nuclei and TDP-43-/NeuN+ from Liu et al. (2019) (*p* = 0.0028, t-test). **d**, Box plot displaying normalized counts of *KALRN* in TDP-43+/NeuN+ nuclei and TDP-43-/NeuN+ from Liu et al. (2019) (*p* = 0.0088, t-test).
